# Subacute Infective Endocarditis Misdiagnosed as Polymyalgia Rheumatica: A Case Report

**DOI:** 10.7759/cureus.94516

**Published:** 2025-10-13

**Authors:** Zorab Rahim, Ali Eftekhar

**Affiliations:** 1 General Medicine, Aneurin Bevan University Health Board, Cwmbran, GBR

**Keywords:** immunosuppression, polymyalgia rheumatica, potential pitfall for misdiagnosis, subacute infective endocarditis, viridans streptococci

## Abstract

Infective endocarditis (IE) is a rare disease with a myriad of signs and symptoms that overlap with other systemic inflammatory disorders. We present the case of an otherwise healthy 57-year-old male patient with a three-month history of back pain, muscle weakness, and weight loss that was initially misdiagnosed as polymyalgia rheumatica (PMR), when in fact he had been suffering from subacute IE due to* *viridans streptococci. The patient’s diagnosis was delayed until there was clinically apparent valve destruction. Our case highlights the crucial importance of excluding clinically subacute bacterial infection in patients with vague constitutional and musculoskeletal symptoms, especially before commencing immunosuppression.

## Introduction

Polymyalgia rheumatica (PMR) is a common inflammatory disorder in adults older than 50 years, with an incidence of 95.9 per 100,000 in the United Kingdom [1]. It is characterised by the presence of proximal hip and/or shoulder girdle stiffness and elevated acute-phase reactants [2] and remains a diagnosis of exclusion, particularly of infection. Misdiagnosis carries significant risk, as initiating immunosuppression in an unrecognised infection can be fatal. Clinicians must therefore remain alert to mimics, with subacute infective endocarditis (IE) representing a notable diagnostic pitfall.

Although far less common with an incidence of up to 10 per 100,000 [3], the protean manifestations of subacute IE in the indolent phase of the disease may overlap with PMR. Constitutional symptoms of weight loss, anorexia, and myalgias are not uncommon and can exist in up to 44% of cases of IE [4]. When IE masquerades as PMR, diagnostic delay risks catastrophic outcomes, including valvular destruction, systemic embolisation, and sepsis. Despite its rarity, mortality remains high, with up to 30% at 30 days [5]. 

We present the case of a patient with no prior risk factors for IE and a three-month-long history of vague constitutional and musculoskeletal symptoms that were initially misdiagnosed as PMR, when in fact he had been suffering from subacute IE. 

## Case presentation

A 57-year-old previously fit and well male bricklayer with no significant past medical history was referred to the rapid diagnostic clinic in March 2023 on the basis of urgent suspected cancer. This was due to a three-month history of episodic severe lower back pain, unintentional weight loss of 5 kg due to loss of appetite, subjective muscle weakness in all four limbs, and persistent fatigue. The patient denied fevers or night sweats. On examination, the patient demonstrated normal power (Medical Research Council (MRC) Scale for Muscle Strength 5/5) both proximally and distally, with no evidence of fasciculations and normal reflexes. No documented examination of the cardiorespiratory system was available. A panel of laboratory investigations was requested (see Tables 1-2), which revealed evidence of a chronic inflammatory disease with a raised erythrocyte sedimentation rate (ESR) and C-reactive protein (CRP) and decreased albumin and inflammatory anaemia. A CT of the thorax, abdomen, and pelvis showed a solitary intrapulmonary lymph node but no evidence of malignancy; images were not available.

**Table 1:** Initial laboratory investigations ANCA: antineutrophil cytoplasmic antibody; PSA: prostate-specific antigen; MPO: myeloperoxidase; PR3: proteinase 3; Ag/Ab: antigen/antibody

Investigation	Normal range	Result
Urine dip	-	Blood ++ (microscopic haematuria)
PSA (ug/L)	<3.6	1.2
Protein electrophoresis	-	No paraprotein detected
Free light chain ratio	0.260-1.650	0.746
B12 (ng/L)	180-900	365
Folate (ug/L)	>3.0	2.2
Haptoglobin (g/L)	0.40-2.80	1.02
Iron (umol/L)	10-30	2
Transferrin (g/L)	2.0-4.0	1.6
Transferrin saturation (%)	20.0-50.0	5.0
Ferritin (ug/L)	15-300	163
HIV Ag/Ab	-	Negative
Hepatitis B surface antigen (HBsAg)	-	Negative
Hepatitis C antibody (anti-HCV)	-	Negative
ANCA screen	-	Positive (titre 1:20)
Anti-MPO antibody (u/mL)	0.0-5.0	2.6
Anti-PR3 antibody (u/mL)	<20.0	17.8

**Table 2:** Inflammatory markers and their progression through the illness Hb: haemoglobin; ESR: erythrocyte sedimentation rate; CRP: C-reactive protein

Investigation	Normal range	At the time of assessment at the rapid diagnostic clinic (three months after onset)	After the first tapering course of oral prednisolone (five months after onset)	At the time of admission to the medical assessment unit (six months after onset)
Hb (g/L)	115-165	94	85	67
White blood cell count (× 10^9^/L)	4.0-11.0	10.9	12.5	14.9
ESR (mm/hr)	0-35	104	83	-
CRP (mg/L)	<10	86	98	111
Albumin (g/L)	35-50	28	24	23

Given the diagnostic uncertainty and signs of an inflammatory illness with no obvious source of infection or evidence of malignancy, a rheumatology opinion was sought. It was ascertained during the rheumatology consultation that the muscle weakness mainly involved the pelvic and shoulder girdles. Further examination of the joints and lymphatic system was normal; again, however, an examination of the cardiorespiratory system was not documented in the patient’s records. A provisional diagnosis of PMR was made, and the patient was commenced on a tapering course of oral prednisolone starting at a dose of 15 mg once daily with a reduction by 2.5 mg every three weeks. An initial slight improvement in only energy and appetite levels was noted. Repeat blood tests showed that despite oral steroids, inflammatory markers continued to be elevated, and a decision was made to give 120 mg of intramuscular methylprednisolone.

**Figure 1:**
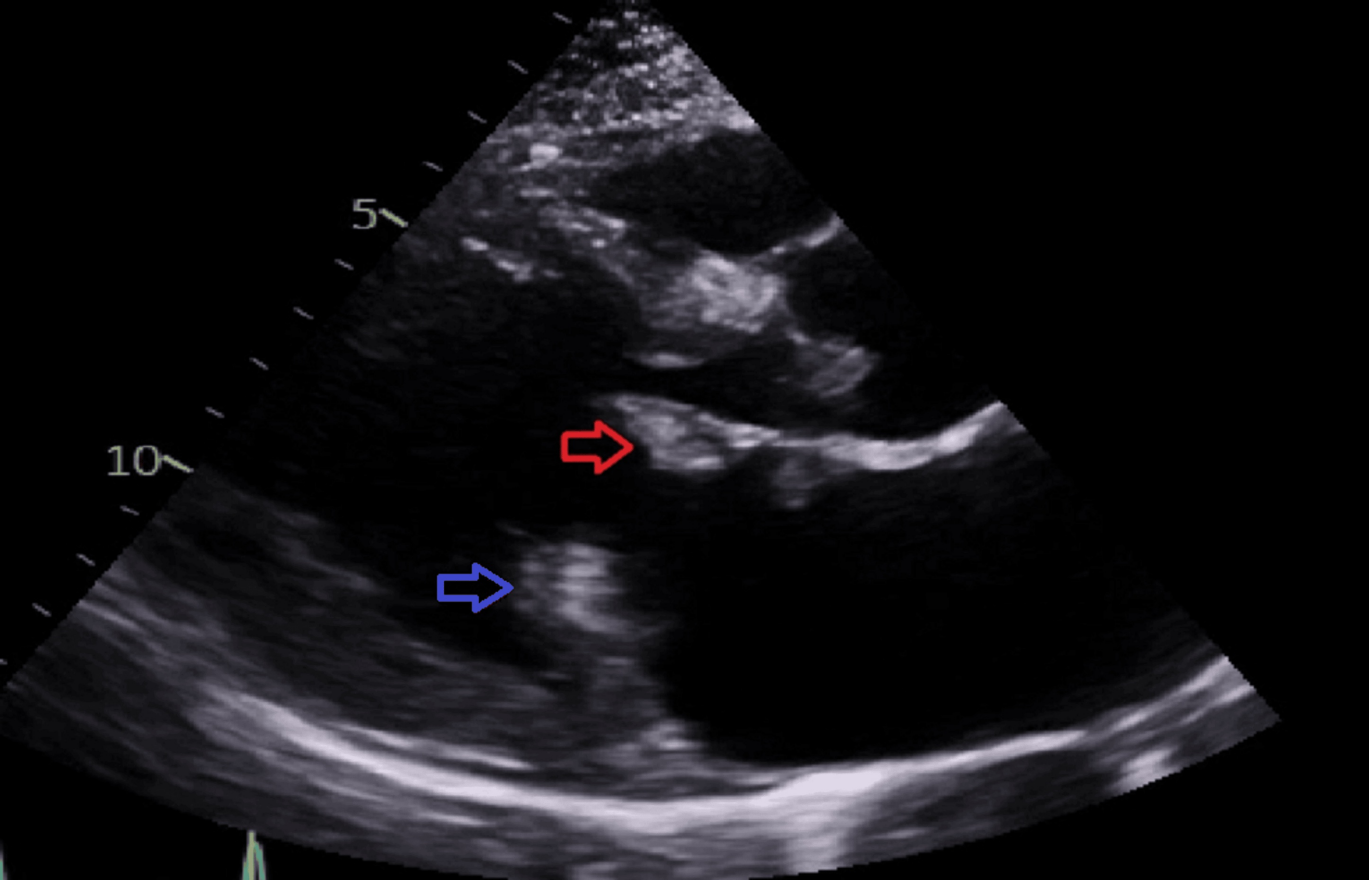
Echocardiogram showing PLAX view of the heart A large (1.4 × 2.1 cm), mobile mass attached to the mid-portion of the atrial surface of the anterior mitral valve leaflet is seen (red arrow). A smaller, mobile mass on the tip of the posterior mitral valve leaflet is also noted (blue arrow). PLAX: parasternal long-axis

A month later, the patient’s anaemia and inflammatory markers continued to worsen, and the patient was admitted to the hospital for further investigations. On admission to the medical assessment unit, the systems review was negative for a gastrointestinal (GI) source of bleeding, and there were no features of infection in the history. Vital observations were as follows: heart rate 119 bpm, respiratory rate 18 bpm, blood pressure 131/77 mmHg, temperature 37.6 degrees Celsius, and oxygen saturations 100% on room air. On physical examination, a loud pansystolic murmur heard loudest at the apex and radiating to the axilla was appreciated; however, no other features of heart failure were evident. A bedside point-of-care echocardiogram (Figure 1) confirmed the presence of large vegetations on the aortic and mitral valves with severe mitral regurgitation and preserved left ventricular (LV) ejection fraction (>65%). 

Alpha-haemolytic streptococci were cultured on three sets of blood cultures, with growth on one culture being identified as *Streptococcus parasanguis*. A definitive diagnosis of subacute IE was therefore made based on meeting the two major Duke’s criteria [6]. Empirical antibiotics were started immediately according to local guidelines, and the patient was transferred under the care of cardiology. Further imaging identified cerebral and splenic emboli, and the patient underwent urgent mechanical aortic and mitral valve replacement. Following several weeks of inpatient antibiotics and cardiac rehabilitation, the patient made a full recovery and was discharged home. At follow-up two years later, the patient remained clinically stable with no features of systemic inflammation or residual rheumatological symptoms. 

## Discussion

Subacute IE can mimic PMR and other inflammatory rheumatic diseases due to overlapping musculoskeletal and constitutional features, which can complicate diagnosis and delay treatment with potentially fatal consequences. 

In Churchill et al.’s retrospective review of 192 cases of bacterial endocarditis, 84 patients had musculoskeletal manifestations, most commonly arthralgias (32), arthritis (26), and low back pain (24) [4]. Similarly, González-Juanatey et al. reported rheumatic features in 46 of 110 episodes of IE, with over half of these patients having underlying cardiac or other predisposing conditions [7]. Our patient was unusual in that he was afebrile, had no predisposing risk factors, and presented predominantly with low back pain - seen in only 12.7% of cases in González-Juanatey et al.’s series. Myalgias, the patient's secondary complaint, were described in 15.5% of cases [7]. These data suggest that while musculoskeletal symptoms occur in IE, they are neither sensitive nor specific markers of disease. 

A prospective cohort of 2781 patients with definite IE found that fever >38°C (96%), raised CRP (62%), raised ESR (61%), and a new murmur (48%) were the most frequent features [8]. Raised CRP and ESR in the absence of a clear source should be alarm signs that prompt consideration of occult bacterial infection, including IE, which can follow a prolonged subacute course, typically without fever. Importantly, the absence of a murmur does not exclude IE. The current 2022 European Alliance of Associations for Rheumatology (EULAR) guidelines do not specifically recommend routine screening for bacterial infection other than tuberculosis [9]. However, in patients with inexplicably raised inflammatory markers and atypical symptoms, repeat blood cultures should be considered to rule out bacteraemia. 

The prolonged subacute phase in this case was also attributable to the infecting organism. Viridans streptococci, such as *Streptococcus parasanguinis*, are commonly associated with late-presenting IE. N’Guyen et al. reported that late-diagnosed IE accounted for one-quarter of all cases and was often linked to nonvirulent streptococci on native valves, with presentations dominated by nonspecific features such as weight loss and asthenia [10]. This fits with our patient’s course, where vague musculoskeletal symptoms delayed consideration of IE until advanced disease with valve destruction was evident. 

The diagnosis of PMR is clinical and one of exclusion. Features that should raise suspicion of an alternative diagnosis include presentation before age 65 [11], normal inflammatory markers (though up to 20% of PMR cases may have normal results), and nonresponse to low-dose steroids [12]. Our patient’s modest improvement in appetite and energy levels on steroids was most likely due to glucocorticoid-induced systemic effects, rather than the resolution of an inflammatory process. The lack of a documented cardiovascular exam at any point in this patient's case before their admission to the hospital and the escalation of their steroid dose despite a poor response in ESR and CRP to low-dose steroids were significant mistakes that led to a delayed presentation of IE until fulminant valve failure.

## Conclusions

It is important for clinicians to bear in mind that PMR is a diagnosis of exclusion. At minimum, a thorough history and full physical examination are required to exclude bacterial causes of inflammation, with the additional consideration of serial blood cultures where diagnostic uncertainty remains. Persistently raised inflammatory markers and lingering musculoskeletal symptoms despite steroids should alert the clinician to a diagnosis other than PMR. The ‘textbook’ signs and symptoms of IE may not be clinically apparent until there is fulminant valve destruction, and this may well occur in patients without prior risk factors for IE. The above case serves as an important reminder of the pitfalls in the diagnosis of PMR and to have a low threshold of suspicion for IE in the constitutionally unwell patient with elevated inflammatory markers. 

## References

[1] Partington RJ, Muller S, Helliwell T, Mallen CD, Abdul Sultan A (2018). Incidence, prevalence and treatment burden of polymyalgia rheumatica in the UK over two decades: a population-based study. Ann Rheum Dis.

[2] Ozen G, Inanc N, Unal AU (2016). Assessment of the new 2012 EULAR/ACR clinical classification criteria for polymyalgia rheumatica: a prospective multicenter study. J Rheumatol.

[3] Rajani R, Klein JL (2020). Infective endocarditis: a contemporary update. Clin Med (Lond).

[4] Churchill MA Jr, Geraci JE, Hunder GG (1977). Musculoskeletal manifestations of bacterial endocarditis. Ann Intern Med.

[5] Delgado V, Ajmone Marsan N, de Waha S (2023). 2023 ESC guidelines for the management of endocarditis. Eur Heart J.

[6] Fowler VG, Durack DT, Selton-Suty C (2023). The 2023 Duke-International Society for Cardiovascular Infectious Diseases criteria for infective endocarditis: updating the modified Duke criteria. Clin Infect Dis.

[7] González-Juanatey C, González-Gay MA, Llorca J (2001). Rheumatic manifestations of infective endocarditis in non-addicts: a 12-year study. Medicine (Baltimore).

[8] Murdoch DR, Corey GR, Hoen B (2009). Clinical presentation, etiology, and outcome of infective endocarditis in the 21st century: the international collaboration on endocarditis - prospective cohort study. Arch Intern Med.

[9] Fragoulis GE, Nikiphorou E, Dey M (2023). 2022 EULAR recommendations for screening and prophylaxis of chronic and opportunistic infections in adults with autoimmune inflammatory rheumatic diseases. Ann Rheum Dis.

[10] N'Guyen Y, Duval X, Revest M (2017). Time interval between infective endocarditis first symptoms and diagnosis: relationship to infective endocarditis characteristics, microorganisms and prognosis. Ann Med.

[11] Bird HA, Esselinckx W, Dixon AS, Mowat AG, Wood PH (1979). An evaluation of criteria for polymyalgia rheumatica. Ann Rheum Dis.

[12] Siebert S, Lawson TM, Wheeler MH, Martin JC, Williams BD (2001). Polymyalgia rheumatica: pitfalls in diagnosis. J R Soc Med.

